# Multiprofessional care promotes of quality of life in pregnant women with preeclampsia: a cross-sectional study

**DOI:** 10.6061/clinics/2020/e1951

**Published:** 2020-11-02

**Authors:** Michelle de Souza Rangel Machado, Tawana Vicente Bertagnolli, Eduardo Carvalho de Arruda Veiga, Cristine Jorge Homsi Ferreira, Geraldo Duarte, Jackeline de Souza Rangel Machado, Ricardo Carvalho

**Affiliations:** I Departamento de Ginecologia e Obstetricia, Faculdade de Medicina de Ribeirao Preto da Universidade de Sao Paulo (FMRP-USP), SP, BR; II Departamento de Biomecanica, Medicina e Reabilitacao do Aparelho Locomotor da Faculdade de Medicina de Ribeirão Preto da Universidade de Sao Paulo (FMRP-USP), SP, BR

**Keywords:** Preeclampsia, Pregnancy, Quality of Life, WHOQOL-Bref, Healthy Pregnancy

## Abstract

**OBJECTIVES:**

To assess the quality of life of hospitalized pregnant women with preeclampsia (PE), and compare with a group of healthy pregnant women (HP).

**METHODS:**

This was an observational cross-sectional study conducted among 58 pregnant women; 28 of them had preeclampsia and 30 were healthy. The WHOQOL-Bref questionnaire, which was divided into four aspects: physical, psychological, social, and environmental, was applied to each subject.

**RESULTS:**

A statistically significant difference was observed regarding maternal age (PE 27.8±6.2 x HG 23.0±6.6, *p*<0.01) and gestational age (PE 224±28.1 x HG 253.8±43.7, *p*<0.01) in relation to the clinical and obstetric data. No significant difference was observed among groups in the physical (PE 57.7±18.9 x HG 65.7±16.6, *p*=0.19), psychological (PE 68.2±12.8 x HG 73.3±13.30, *p*=0.16), social (PE 72.0±15.8 x HG 71.7±18.7, *p*=0.78), or environmental (PE 61.1±11.9 x HG 59.3±15.9, *p*=0.88) aspects of the WHOQOL-Bref.

**CONCLUSION:**

There was no difference in quality of life between the groups studied, a result possibly due to the fact that women with PE were hospitalized and received multiprofessional care.

## INTRODUCTION

In a 2014 survey, the World Health Organization reported that the causes of maternal death are related to pre-existing morbidities (28%) severe hemorrhages (27%), pregnancy-induced hypertensive diseases (14%), infections (11%), complications during delivery (9%), abortion (8%), and coagulopathies (3%) (1,2).

Hypertensive disorders of pregnancy affect approximately 6 to 8% of all pregnant women worldwide, contributing significantly to severe maternal-fetal complications (3). Preeclampsia affects approximately 3-5% of all pregnancies, and it is one of the major causes of maternal and perinatal morbidity and mortality worldwide, being currently responsible for about 60,000 maternal deaths, especially in poor countries (4-6).

Few studies have investigated the physical, emotional, and psychological aspects of preeclampsia in pregnant women and the extent to which they may affect the quality of life of these women (7).

The World Health Organization defines quality of life as the perception a person has of their own life within the context of their culture and values, and personal objectives, standards, and concerns (8). During the gestational period, the women’s standards and perception of life may change since the focus is on the fetus during this period. Therefore, women need to go through psychological adaptation due to concerns and fears for the pregnancy, which may affect the quality of life (9).

Studies have reported that when the physical performance and the perception of the level of health and well-being of a woman were compared before and during pregnancy, it was observed that these levels were reduced during pregnancy (10,11). Although most of the physical changes occurring during pregnancy are reversed within 8 weeks of delivery, women may experience many physical and mental symptoms related to delivery during this critical period (12). After being diagnosed with risky pregnancy and PE and during hospitalization, these women have a better perception of their disease and they start to question themselves about what led to their current condition. This may generate a feeling of guilt and anxiety, with a consequent change in their quality of life (13-16).

The objective of the present study was to assess the quality of life of hospitalized pregnant women with preeclampsia and compare it to that of healthy pregnant women.

## MATERIAL AND METHODS

This was a cross-sectional observational study conducted among hospitalized pregnant women diagnosed with preeclampsia or hypertension with superimposed preeclampsia and among healthy pregnant women. The study was approved by the Research Ethics Committee of the University Hospital, Faculdade de Medicina de Ribeirão Preto, Universidade de São Paulo (HCFMRP-USP) with the protocol approval number 6500/2010, and all subjects gave written informed consent to participate. This study was conducted at the high-risk pregnancy ward of the HCFMRP-USP and at the MATER referral women’s health center of Ribeirão Preto (Centro de referência da saúde da mulher de Ribeirão Preto), where the women were invited to participate in the investigation.

### Inclusion criteria

The patients were admitted to control their blood pressure, laboratory tests, and fetal vitality (patients with difficulty in controlling blood pressure with three drugs, worsening laboratory tests, critical evolution of fetal vitality, and signs of impending eclampsia, due to the severity of the condition, were excluded from the evaluation). Patients with chronic arterial hypertension with preeclampsia (indicated by proteinuria ≥0.3 grams/24 hours and blood pressure ≥140/90 mmHg) (NHBPEP, 2000) or chronic hypertension with superimposed preeclampsia (indicated by proteinuria ≥0.3 grams/24 hours), as it did not occur before the 20th week and between 24 and 38 gestational weeks, admitted to the Gynecology and Obstetrics ward; pregnant women with a confirmed diagnosis of preeclampsia since the 24th gestational week who had been admitted to the high-risk pregnancy ward of HCFMRP-USP (PE group); and healthy pregnant women (HP group) with no diseases since the 24th gestational week and seen at the MATER outpatient clinic were included in this evaluation. The authors also included patients diagnosed with preeclampsia and those with hypertension who developed preeclampsia after 20 gestational weeks.

### Exclusion criteria

Patients were excluded if they were had:

1. Multiple gestations.
2. Fetal suffering diagnosed by examining fetal vitality for those who were symptomatic (clouded vision, epigastralgia, and headache).
3. Laboratory changes (thrombocytopenia, altered renal function, elevated liver enzyme and bilirubin levels).
4. Gestational diabetes.
5. Maternal heart disease and ultrasound findings of altered fetal morphology.

Twenty-eight patients per group (PE and HP) were recruited. Quality of life was assessed with the WHOQOL-Bref questionnaire, which evaluates the general quality of life of patients, and the questionnaire was validated by the literature. The WHOQOL-Bref questionnaire has four main characteristics: Domain of general quality of life, physical domain, psychological domain, and domain of personal relationships (17). The WHOQOL-Bref contains 26 questions, two of them regarding quality of life in general and the remaining 24 regarding 4 domains: physical, psychological, social relations, and environment quality of life, which were assessed using a positive scale, *i.e.*, the higher the score, the better the quality of life. Since there are no cut-off points, the scores were divided into 5 from a total (100) score; the replies were given using a 5-item Likert scale, which is a type of psychometric response scale that specifies the level of agreement with the statement. Thus, the quality of life scores were given as 0-20, very poor; 21-40, poor; 41-60, neither bad nor good; 61-80, good; and 81-100, very good (18).

### Statistical analysis

The clinical and WHOQOL-Bref data were subjected to descriptive analysis. The Mann-Whitney test was used to compare the groups according to age, parity, gestational age, and body mass index (BMI); analysis of covariance (ANCOVA) was used to compare the groups according to the domains of the WHOQOL-Bref. The analyses were carried out using the PROC MIXED feature of the SAS^®^ 9.0 software, with the significance level set at *p* <0.05.

## RESULTS

The characterization of PE and HP subjects by age, parity, gestational age, and BMI is presented in Table 1. A statistically significant difference between groups was observed only in gestational age, with the healthy pregnant women being younger and having higher gestational age than those in the PE group.

The mean scores for each domain of the WHOQOL-Bref questionnaire are presented in Table 2, showing no difference between the groups. However, PE subjects showed neither good nor poor quality of life only in the physical domain, while HP subjects showed neither good nor poor quality of life only in the environmental domain. The remaining domains of the questionnaire revealed good quality of life for the pregnant women in both groups.

Table 3 presents a comparison of the age, parity, and gestational age and BMI in relation to the scores for the domains of the WHOQOL-Bref. A significant difference was observed between the environmental domain and age (*p*=0.04) and between the environmental domain and parity (*p*=0.04).

## DISCUSSION

In this study, after comparing the groups (PE and HP) according to age, parity, gestational age, and BMI, we observed that HP women were younger than women with PE and there were no differences in the quality of life between the groups. Additionally, HP women had higher gestational age, as they were recruited at a low-risk gestation unit.

However, a limitation of this study was the fact that there was no specific questionnaire for quality of life assessment in pregnant women. In addition, only a few studies have assessed the quality of life of pregnant women with preeclampsia. In contrast to normal pregnancy, pregnancy with preeclampsia involves a series of complications that may cause physical as well as psychological discomfort to the patient, and in many cases, would require hospitalization. In addition, depending on its severity, the disease may pose a risk not only to the mother but also to the fetus (10,13,14,18). In the multivariate analysis (ANCOVA), the domains of the WHOQOL questionnaire were considered as outcomes, while age, parity, gestational age, BMI, and groups were considered as covariates. In this analysis, we verified that there was an effect of age and parity, according to the estimated coefficient, in only the environmental domain; age increased and parity decreased as the environmental domain scores increased (Table 3).

Previous studies associated quality of life according to the WHOQOL-Bref questionnaire, with pregnancy and several aspects related to it. For example, a study linked worse quality of life results in women with preeclampsia, while another study found favorable quality of life results in pregnant women who performed physical activities and worse results for those who had depression and insomnia during pregnancy, and a third study showed worse quality of life results in diabetic pregnant women (19-21).

Based on this principle, we may infer that women with PE would have a worse quality of life compared to other pregnant women. However, in our study we did not observe a difference in quality of life between the groups, as the mean values regarding the domains of the WHOQOL were similar. This may be because women with PE received multiprofessional care during hospitalization. This kind of care may have been crucial for women with PE to give a positive response to the questionnaire regarding quality of life, since they received greater attention from the doctors and nurses, as well as psychological counseling; they received assistance with more attention because their pregnancy was considered as high-risk. These results agree with those reported by Rezende and Souza (22); they assessed the quality of life of high-risk pregnant women during a medical visit and concluded that, despite their physical and emotional discomfort during pregnancy, their quality of life was positive.

Stern et al. (23) evaluated that pregnant women with severe preeclampsia, assisted by a specialized and well-trained multidisciplinary team, can generally have positive outcomes regarding the quality of life of the mother and the neonate because of the favorable reception. In a 2014 study, Mortazavi et al. (24) investigated pregnant women in their third trimester and during their postpartum period. The quality of life results during their third trimester and postpartum period, respectively, were as follows: very good (27%, 26.5%), good (54%, 52.8%), neither good nor poor (18%, 19%), poor (0.8%, 0.9%), and very poor (0.3%, 0.9%). These data show that most of the pregnant women had a reasonable quality of life during both periods. In the present study, we observed that women in the PE and HP groups had a good general quality of life.

Additionally, in a study in which 42 pregnant women were assessed using the WHOQOL-Bref, it was observed that the subjects had a good general quality of life. It also demonstrated that the WHOQOL-Bref is pertinent as a monitoring instrument, thus permitting the expansion of prenatal care (16). In this study, we also used the WHOQOL-Bref since there is no specific questionnaire for assessing the quality of life of pregnant women.

Ferreira et al. (25) applied the WHOQOL-Bref questionnaire to 51 low-risk pregnant women in their second trimester, using only the first question of the instrument “How would you assess your quality of life?” They observed that that most of the subjects reported a good quality of life.

The results of another study in which the quality of life and of sleep were assessed in 100 pregnant women at 15-25 gestational weeks revealed a significant relationship between the two indices. This scientific work indicated that the quality of life of pregnant women was directly related to their quality of sleep since both indices decrease with increasing gestational age (26,27).

Bień al. (27) reported that diseases during pregnancy significantly affect the life style and psychological well-being of the patients, contributing to increased stress and consequently impairing their quality of life. In the present study, we observed that diseases did not affect the quality of life of the patients with preeclampsia because they received full hospital and multiprofessional support throughout their hospitalization period (26). In a single study, the authors assessed the association of hypertension with the quality of life in hypertensive and healthy pregnant women along the trimesters before the prenatal care visits, using the Ferrans & Powers Quality of Life Index questionnaire. They observed that hypertension was associated with poor quality of life since the hypertensive patients had lower quality of life scores than the normotensive ones had (28).

## CONCLUSIONS

The expectation of the present study was that patients with preeclampsia would have worse quality of life scores due to hospitalization and the complications of the disease. However, when we applied the WHOQOL questionnaire to these patients we observed the contrary, *i.e.*, the pregnant women with preeclampsia had good quality of life scores just like the healthy controls. This may be explained by the care received by these patients from a multidisciplinary team. We may conclude that receiving multiprofessional and specialized care may reflect on good quality of life.

## HIGHLIGHTS

Preeclampsia affects 2 to 3% of all pregnancies worldwide, with a high rate of maternal and perinatal morbidity and mortality, high costs for the health system and high hospitalization rates, which may affect the quality of life of these women

## AUTHOR CONTRIBUTIONS

Machado MSR participated in the design of the study, data collect, performed the statistical analysis, and drafted the manuscript.

Bertagnolli TV participated in the design of the study and data collection.

Ferreira CJH participated in the design of the study and drafted the manuscript.

Duarte G drafted the manuscript.

Veiga ECA performed the statistical analysis and drafted the manuscript.

Machado JSR performed the statistical analysis and drafted the manuscript.

Carvalho R conceived of the study and participated in its design and coordination, and helped to draft the manuscript.

All authors read and approved the final manuscript.

## Figures and Tables

**Table 1:** Characteristics of the pregnant women with preeclampsia (PE) and of the control healthy pregnant women (HP).

Parameters	PE (n=28)	HP (n=30)	*p*-value
Age	27.8±6.2	23.0±6.6	**<0.01**
Parity	2.2±1.6	1.7±0.9	0.28
Gestational age (days)	224.0±28.1	253.8±43.7	**<0.01**
BMI	31.2±5.9	29.8±5.7	0.44

BMI, body mass index. Values are reported as means ±SD.

**Table 2:** Comparison of the mean scores for the domains of the WHOQOL-Bref questionnaire between pregnant women with preeclampsia (PE) and healthy pregnant women (HP).

Paramteres	PE (n=28)	HP (n=30)
WHOQOL-Physical	57.65±18.9	65.71±16.6
WHOQOL-Psychological	68.16±12.8	73.75±13.3
WHOQOL-Social	72.02±15.8	71.67±18.7
WHOQOL-Environmental	61.05±11.9	59.27±15.9

WHOQOL (Quality of life questionnaire of the World Health Organization). Values are reported as means ±SD.

**Table 3:** WHOQOL-Bref Questionnnaire: comparison of the variables with facets.

WHOQOL-physical				
**Variable**	**Estimated coefficient**	***p*-value**	**95%CI**	
Age	-0.19	0.67	-1.08	0.70
Parity	-2.00	0.36	-6.34	2.35
GA (days)	-0.05	0.62	-0.23	0.14
BMI	-0.72	0.09	-1.56	0.11
**Comparison**	**Estimated coefficient**	***p*-value**	**95%CI**	
Preeclampsia *vs* Control	-7.79	0.19	-19.60	4.02

WHOQOL-psychological				
**Variable**	**Estimated coefficient**	***p*-value**	**95%CI**	
Age	0.43	0.21	-0.25	1.11
Parity	-1.76	0.29	-5.07	1.55
GA (days)	0.01	0.84	-0.13	0.15
BMI	-0.33	0.31	-0.97	0.31
**Comparison**	**Estimated coefficient**	***p*-value**	**95%CI**	
Preeclampsia *vs* Control	-6.43	0.16	-15.43	2.56

WHOQOL-social				
**Variable**	**Estimated coefficient**	***p*-value**	**95%CI**	
Age	-0.42	0.34	-1.30	0.46
Parity	-2.66	0.22	-6.95	1.63
GA (days)	-0.08	0.38	-0.26	0.10
BMI	-0.28	0.49	-1.11	0.54
**Comparison**	**Estimated coefficient**	***p*-value**	**95%CI**	
Preeclampsia *vs* Control	1.60	0.78	-10.06	13.27

WHOQOL-environmental				
**Variable**	**Estimated coefficient**	***p*-value**	**95%CI**	
Age	0.73	0.04	0.03	1.43
Parity	-3.46	0.04	-6.87	-0.05
GA (days)	0.03	0.69	-0.12	0.17
BMI	-0.38	0.26	-1.04	0.28
**Comparison**	**Estimated coefficient**	***p*-value**	**95%CI**	
Preeclampsia *vs* Control	0.70	0.88	-8.57	9.97
